# DNA double-strand break repair and nucleic acid-related immunity: DSB repair and nucleic acid-related immunity

**DOI:** 10.3724/abbs.2022061

**Published:** 2022-06-01

**Authors:** Haiping Zhang, Yu Chen, Ying Jiang, Zhiyong Mao

**Affiliations:** Shanghai Key Laboratory of Maternal Fetal Medicine Clinical and Translational Research Center of Shanghai First Maternity and Infant Hospital Frontier Science Center for Stem Cell Research School of Life Sciences and Technology Tongji University Shanghai 200092 China

**Keywords:** innate immune, double-strand break repair, nucleic acid sensor, human disease

## Abstract

DNA damage repair and innate immunity are two conserved mechanisms that both function in cellular stress responses. Recently, an increasing amount of evidence has uncovered the close relationship between these two ancient biological processes. Here, we review the classical function of factors involved in DNA repair, and especially double-strand break repair, in innate immunity; more importantly, we discuss the novel roles of DNA repair factors in regulating innate immunity and *vice versa*. In addition, we also review the roles of DNA repair, innate immunity and their crosstalk in human diseases, which suggest that these two pathways may be compelling targets for disease prevention and treatment.

## Introduction

Genomic integrity maintenance is essential for the growth, development and survival of organisms. However, the genome is exposed to exogenous and endogenous insults such as genotoxic chemicals, ionizing radiation, ultraviolet light, stalled replication forks, and reactive oxygen species (ROS) generated during mitochondrial oxidative metabolism, which can generate several different kinds of DNA lesions [ 1, 2] . In addition, the processes of V(D)J recombination and class switch recombination, which are both important events contributing to immunoglobulin diversity, are also mediated by programmed DNA damage [ 3, 4] . Among all types of DNA lesions, double-strand breaks (DSBs) are considered to be the most serious, and they can be repaired by two independent and competing pathways, namely, homologous recombination (HR) and nonhomologous end joining (NHEJ) [ 5, 6] . A large number of factors are involved in regulating the different steps in HR and NHEJ, and it has been reported that loss of function of DSB repair factors can give rise to severe diseases such as cancer and premature aging syndromes.

Innate immunity is responsible for sensing the presence of exogenous nonself- or endogenous self-derived nucleic acids to trigger a series of responses, including attacking foreign pathogens, clearing apoptotic cells and promoting tissue repair [ 7, 8] . Recognizing cytosolic nucleic acids is the core step of innate immune signaling, and a variety of nucleic acid sensors have been identified to function in different contexts. Importantly, in recent years accumulating evidence has positioned the cytosolic nucleic acid sensing pathway as the key link between innate immunity and the DNA damage response [ 9– 11] . The function of innate immune signaling in the DNA damage response and the potential role of immune regulation in the DNA repair process are of great interest for interdisciplinary research.

In this review, we discuss the classical and nonclassical functions of DNA repair factors and innate immune regulators, and their potential therapeutic value in treating human diseases.

## DNA Damage and DNA Double-strand Break Repair

As mentioned above, HR and NHEJ cooperate to stabilize the genome. HR occurs mainly during the late S/G2 phase because it requires the homologous sequence on the sister chromatid. Upon DSB formation, the MRN (MRE11–RAD50–NBS1) complex and CtIP are recruited to the damage site to initiate the end resection process, followed by EXO1 and BLM–DNA2-mediated long-range resection. Then, RPA quickly binds to single-stranded DNA and is subsequently replaced by RAD51 recombinase in a manner dependent on the activity of the breast cancer-related factors BRCA1 and BRCA2. RAD51 carries damaged single strands to search for homologous DNA strands and finally complete HR repair [12].

NHEJ is active throughout the cell cycle. It can be further classified into two independent subpathways, namely, canonical nonhomologous end joining (c-NHEJ) and alternative NHEJ (alt-NHEJ). In the process of c-NHEJ, the KU70/KU80 heterodimer first binds to the end of the DSB and recruits DNA-PKcs to form the DNA-PK holoenzyme; then, Artemis binds to and is phosphorylated by DNA-PKcs to be activated for the processing of the complexed structure at broken ends. Finally, the XRCC4/DNA LIG4/XLF complex bridges the ends to finish c-NHEJ repair [13]. The main factors involved in the alt-NHEJ pathway include PARP1, XRCC1, LIG3 and POLθ. Alt-NHEJ is an error-prone pathway which depends on microhomologies to join the broken ends. It typically causes the deletions of large DNA fragments [14]. DNA damage repair is crucial for the maintenance of genome stability. Once the balance between the occurrence and repair of DNA damage is disrupted, severe consequences such as insertions, deletions and accumulation of rearrangements will be triggered, eventually leading to cell death, aging and even cancer [15].

## Nucleic Acid Sensors in the Innate Immune Response

**Figure 1:**
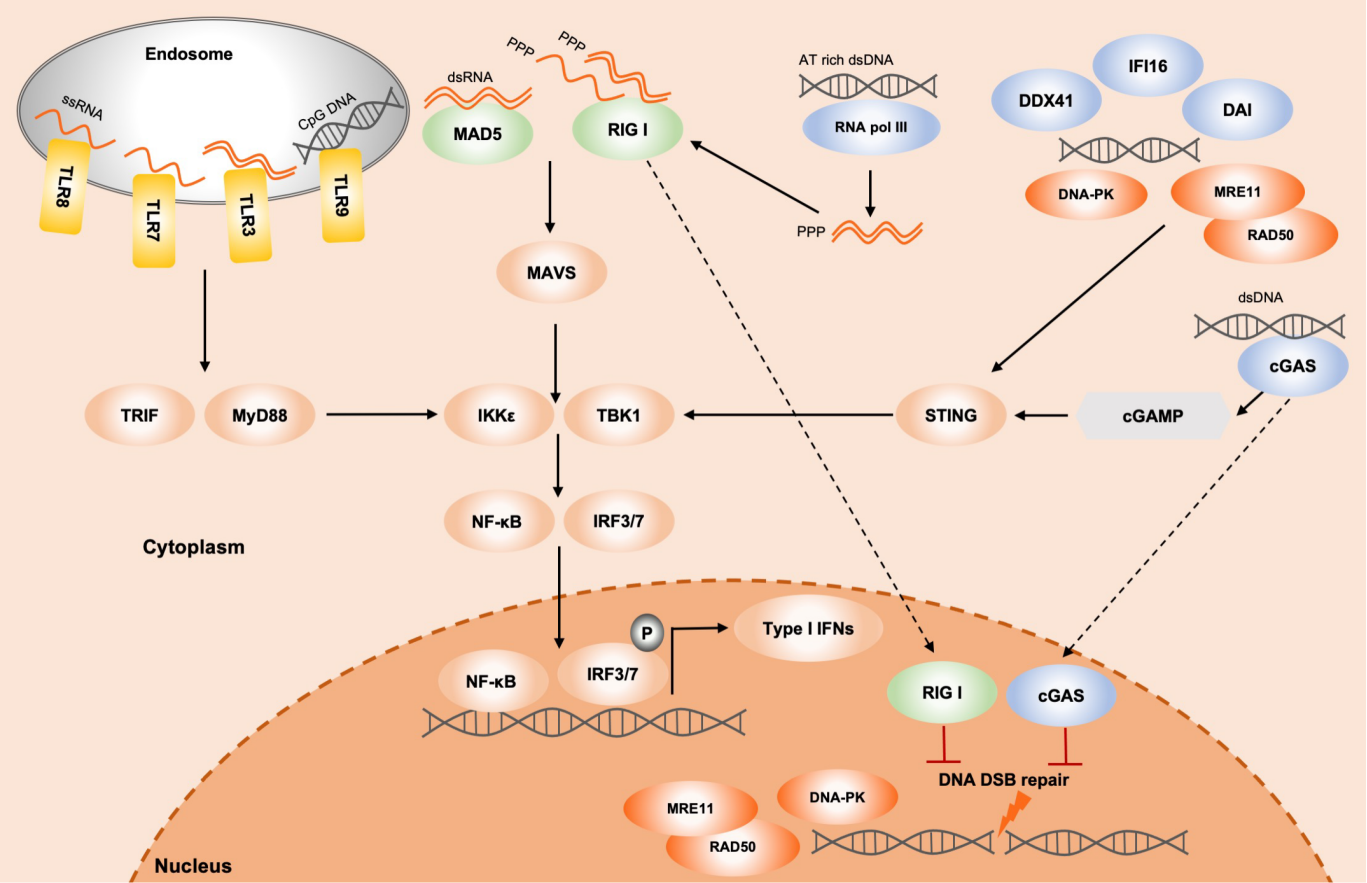
Figure 1 Nucleic acid immunity and DNA double-strand break repair The cytoplasmic nucleic acids are detected by various PRRs. The TLRs include TLR3, TLR7, TLR8 and TLR9, which are located in the endosome. TLR3 detects dsRNA, TLR7/8 detects ssRNA and TLR9 detects unmethylated CpG DNA, to recruit the TRIF or MyD88 and activate the kinases TBK1 and IκB kinase-ε (IKKε). Then TBK1 and IKKε activate the transcription factors IRF3/7 and NF-κB, and finally turn on the expression of type I IFNs. RIG-I detects the uncapped 5’-triphosphate of ssRNA and short dsRNA, while MDA5 recognizes longer dsRNA. Both RIG-I and MDA5 can interact with the mitochondria antiviral signaling protein (MAVS), and then activate the TBK1 and IKKε, eventually turn on the expression of type I IFNs. Cytosolic dsDNA is recognized by cGAS, DAI, DDX41 and IFI16, which can further activate the type I IFNs response through STING–TBK1–IRF3 axis. Several DSB repair factors have been shown to function in sensing nucleic acids. For instance, MRE11/RAD50, both are critical HR factors, and DNA-PK, the core NHEJ complex which consists of KU70, KU80 and the catalytic subunit DNA-Pkcs, can detect the cytosolic dsDNA to trigger type I IFNs through STING–TBK1–IRF3 signaling pathway. Notably, some immune factors have also been shown to be involved in the regulation of DSB repair. For instance, cGAS can translocate into the nucleus and be recruited to DSB sites, leading to the inhibition of HR repair, genomic instability and tumorigenesis. RIG-I has also been demonstrated to be recruited to DSB sites to regulate NHEJ repair by impeding the formation of XRCC4/LIG4/XLF complex, contributing to genomic instability.

Innate immunity is indispensable because it functions as the first line of host defense against various pathogenic invasions and infections. The innate immune responses can be activated by pathogen-associated molecular patterns (PAMPs), such as various antigenic factors of viruses and bacteria, or by damage-associated molecular patterns (DAMPs), which are derived from endogenous damaged and dying cells. The human body has evolved multiple kinds of pattern recognition receptors (PRRs) to recognize PAMPs and DAMPs [ 16– 18] . Nucleic acid sensors are indispensable PRRs that play fundamental roles in the immune response by recognizing bacterial or viral DNA/RNA and nucleic acids derived from the host. There are a variety of DNA and RNA sensors, such as cyclic guanosine monophosphate (GMP)-adenosine monophosphate (AMP) synthase (cGAS), Toll-like receptors (TLRs), NOD-like receptors (NLRs), and retinoic acid-inducible gene I (RIG-I) like receptors (RLRs) [19] ( Figure 1).

DNA sensors include TLR9 [20], DNA-dependent activator of interferon (IFN) regulatory factor (DAI) [21], interferon gamma-inducible protein 16 (IFI16) [22], RNA polymerase III [23], DDX41 [24], AIM2 [25] and cGAS [26]. Different DNA sensors are responsible for recognizing different types of DNA. TLR9 is the only receptor known to recognize and bind to bacterial DNA that is rich in unmethylated CpG dinucleotides in plasmacytoid dendritic cells and B cells [20]. DAI is the first cytosolic dsDNA sensor discovered to activate the TANK-binding kinase 1 (TBK1)-IFN regulatory factor 3 (IRF3)-type I IFN immune pathway [ 21, 27] . Intriguingly, there is no difference between DAI-deficient mice and wild-type mice in innate immune responses [ 27, 28] . IFI16 is a transcription factor that plays roles in recognizing nuclear pathogens. IFI16 can translocate into the cytoplasm and promote STING–IRF3 and STING–NF-κB pathway, in response to the infections by Kaposi’s sarcoma-associated herpesvirus and herpes simplex virus [ 22, 29] . RNA polymerase III detects AT-rich dsDNA in the cytosol and induces type I IFNs through the RIG-I pathway [23]. DDX41, a member of the DEXDc family of helicases, recognizes cytosolic DNA, including self-DNA and viral DNA, in myeloid dendritic cells (mDCs) and promotes type I IFNs and other cytokine responses in a STING (also known as MITA, MPYS, and TMEM173)-dependent manner [24]. AIM2 can also detect cytoplasmic foreign DNA to initiate inflammasome activation and cell death [ 25, 30, 31] . Another DNA sensor is cGAS, whose discovery was a milestone in immunology. cGAS can detect not only cytosolic dsDNA but also RNA:DNA hybrids [32] and catalyze the formation of the second messenger cyclic guanosine monophosphate-adenosine monophosphate (cGAMP) from ATP and GTP and further activate the adaptor protein STING. STING then recruits TBK1 and IκB kinase to activate IRF3 and NF-κB, finally activating the expression of type I IFNs and other inflammatory cytokines [ 26, 33, 34] . It has been demonstrated that cGAS is essential for the production of type I IFNs against DNA viruses and is expressed in multiple cell types [35]. Moreover, cGAS activation depends on the length of DNA fragments, and longer DNA fragments more easily activate cGAS [36]. Interestingly, a recent study has also shown that cGAS localizes to the plasma membrane through its N-terminus in an inactive state, which avoids identifying self-DNA, while cGAS can be easily activated upon pathogenic microorganisms such as bacteria and viruses invading [37].

RLRs and TLRs are both RNA sensors that recognize and defend against RNA viruses. RLRs localize in the cytosol and include RIG-I, melanoma differentiation-associated protein 5 (MDA5) and laboratory of genetics and physiology 2 (LGP2) [ 38, 39] . RNA-responding TLRs, including TLR3, TLR7, TLR8, and TLR13, are located in the endosome [40]. TLR3 senses double-stranded RNA (dsRNA) independent of the RNA sequence [ 41– 43] , is activated by binding to the adaptor protein TRIF and induces the activation of the TRAF3–TBK1/IKKε–IRF3 axis. Guanosine (G)-rich and uridine (U)-rich single-stranded RNA (ssRNA) is recognized by TLR7 and TLR8, which share high levels of homology [ 44– 46] . TLR7 is mainly expressed in B cells and plasmacytoid dendritic cells, while TLR8 is expressed in monocytes, macrophages and myeloid dendritic cells [ 47, 48] . TLR13 has an appetite for bacterial 23S rRNA [49]. MyD88 is an adaptor protein for TLR7, TLR8, and TLR13, and their interaction is responsible for the activation of the TRAF6–TAK1–MAPKs/NF-κB/IRF5 signaling pathway, which contributes to the expression of type I and III IFNs and proinflammatory cytokines.

## The Role of DSB Repair-related Factors in the Innate Immune Response

The innate immune response is closely associated with DSB formation. Several studies have demonstrated that the formation of DSBs is accompanied by an immune response and inflammation. For instance, ionizing radiation (IR), UV irradiation and the topoisomerase inhibitor etoposide can induce the production of many different kinds of inflammatory cytokines, such as TNF-α and IL-6, in skin and lung cells [ 50– 53] . A study in 2017 provided one possible explanation, that endogenous and exogenous DNA damage results in the formation of micronuclei, and damaged DNA will be exposed to cGAS upon micronuclear envelope breakdown to promote a proinflammatory response [9].

Not only can DNA damage *per se* stimulate the innate immune response, but the factors involved in DSB repair are also involved in the regulation of immunity. Many lines of evidence have shown that some DNA damage sensors can translocate into the cytoplasm to trigger the immune response by sensing foreign DNA. DNA-PK is an NHEJ repair protein complex that consists of KU70, KU80 and the catalytic subunit DNA-PKcs. In addition to its classical role in DNA repair, DNA-PK has been identified as a novel DNA sensor for triggering type I IFN gene transcription through the STING–TBK1–IRF3 signaling pathway in murine embryonic fibroblasts (MEFs) and adult murine skin fibroblasts. DNA-PKcs has been shown to phosphorylate IRF3 upon dsDNA stimulation [54]. Loss of DNA-PKcs contributes to impaired transcription of cytokine genes in MEFs. Interestingly, recent research has shown that DNA-PKcs can activate a STING-independent DNA sensing pathway and inhibit cGAS enzymatic activity by phosphorylating cGAS [ 55, 56] , indicating the existence of cross-talk between different DNA sensing pathways.

KU also participates in the regulation of innate immunity. Mice lacking *Ku70* or *Ku80* exhibit greatly reduced cytokine induction [57]. KU70 has been identified as a novel DNA sensor inducing IFN-λ1 activation [58]. Mechanistic studies have demonstrated that STING is an essential mediator of the KU70-mediated production of IFN-λ1 and that KU70 translocates into the cytoplasm to form a complex with STING upon DNA exposure [59]. Moreover, KU70 cytoplasmic translocation depends on its heterodimerization with KU80, although KU80 is not directly involved in cytosolic DNA sensing [60]. As a DNA sensor, cytoplasmic KU70 in liver-derived cells has been reported to promote hepatitis-associated chemokine secretion by sensing HBV DNA [61].

The MRE11-RAD50-NBS1 (MRN) complex involved in HR is also a dsDNA sensor. MRE11 senses pathogen- or dead cell-derived dsDNA to activate the STING–TBK1–IRF3 cascade, turning on the expression of type I IFNs. A lack of MRE11 in cells will reduce the production of type I IFNs. In addition, RAD50, rather than NBS1, is essential for the recognition of dsDNA and activation of STING [62]. Another study showed that RAD50 also directly interacts with CARD9, an innate immune adapter, and that the dsDNA-RAD50-CARD9 complex is significant for NF-κB activation and pro-IL-1β production in dendritic cells [63]. Moreover, XRCC4, a c-NHEJ factor, has also been shown to suppress RNA virus replication [64].

Taken together, these findings show that DSB repair-related factors play central roles in the regulation of the innate immune response ( Figure 1). It is highly attractive to explore whether there are more DNA repair-related factors mediate the innate immune response. The biological significance underlying the crosstalk between DNA repair and innate immune response is to be elucidated.

## Immune Factors Are Involved in DNA Damage Repair

As mentioned above, DNA sensors such as cGAS are considered critical factors linking DNA damage with inflammation, and the regulatory effect of immunity has been related to the onset of cellular senescence [ 65, 66] . However, molecular evolutionary analysis indicates that cGAS appears earlier than the TBK1 and type I IFNs, which implies that cGAS has other functions in addition to innate immune regulation [ 67, 68] . Recent studies by others and us have uncovered the novel role of cGAS in regulating DNA repair and replication in the nucleus. Upon DSBs, cGAS translocates into the nucleus and is recruited to damage sites [69]. The interaction between cGAS and PARP1 via poly (ADP-ribose) (PAR) impedes the formation of the Timeless/PARP1 complex, thereby inhibiting HR repair and leading to genomic instability and tumorigenesis [69] ( Figure 1). Furthermore, another independent study also demonstrated that cGAS can compact template DNA into a higher-ordered state to resist RAD51-mediated DNA strand invasion and suppress HR repair [70]. The inhibitory effect of cGAS on HR repair does not depend on cGAMP synthase activity [69]. Another recent study showed that cGAMP reduces the availability of NAD ^+^, inhibits PARylation and suppresses HR repair in an IFN-independent fashion [71]. Moreover, cGAS has also been reported to function as a decelerator of replication forks [72]. Collectively, these findings suggest that the nuclear role of cGAS is complex and clearly needs to be further clarified. Elucidating the underlying mechanism regulating the subcellular localization of cGAS will improve our understanding of the balance between its nuclear and cytosolic function.

Surprisingly, the RNA-sensing pathway can also be activated by DNA damage stress [73]. RIG-I, a key cytosolic RNA sensor inducing immune responses to viral infection, can detect dsRNA and single-stranded RNA [ 74– 76] . RIG-I contains two N-terminal caspase activation and recruitment domains (CARDs), a helicase domain and a carboxy-terminal domain (CTD) [77]. RIG-I is naturally located in the cytoplasm in an inactive conformation in the absence of viral RNA [ 78, 79] , while upon viral RNA binding, CARDs undergo conformational changes to interact with the MAVS and then activate TBK1 and IKKε, eventually turning on the expression of type I IFNs [ 80– 82] . Recent studies have shown the nuclear localization of RIG-I [83], and it plays a key role in the regulation of the NHEJ pathway [64]. Mechanistically, RIG-I can be recruited to DSB sites and interact with XRCC4, which impedes the formation of the XRCC4/DNA LIG4/XLF complex and thereby inhibits NHEJ repair, promoting genome instability [64] ( Figure 1).

Although the alteration of the subcellular localization of PRRs has been demonstrated to be one of the core events that induce the role switch of PRRs, the regulatory mechanisms underlying the trade-off between their functions in DNA repair and immune activation clearly need further investigation.

## DSBs Repair Defects and Immune-related Diseases

Immune-related diseases are closely related to DSB repair and genomic integrity [84]. DNA repair defects are often accompanied by the activation of the innate immune response. Many studies have shown that DNA damage-induced expression of type I IFNs may be one of the factors that trigger immune-related diseases.

In humans, ataxia-telangiectasia (A-T) syndrome, a rare genetic disorder characterized by neurodegeneration, cancer predisposition and dysfunction of the immune response, occurs due to mutations in the *ATM* gene [85]. ATM is a protein kinase involved in the DNA damage response that promotes DSB repair. It is mainly activated by interacting with NBS1 of the MRN complex and is the main kinase responsible for phosphorylation of histone H2AX, which occurs rapidly after DSBs and serves as the basis for the assembly of DNA repair machinery [86]. *ATM* deficiency decreases genomic integrity and leads to the accumulation of cytosolic DNA fragments, which can activate the cGAS–STING pathway [ 87, 88] . Consistent with this, *Atm* ^−/−^ mice present enhanced antiviral and antibacterial responses with induced type I IFN production, and this autoinflammatory phenotype can be alleviated by cGAS knockdown [89]. These studies imply that boosting DSB repair and blocking the activity of nucleic acid sensors have potential therapeutic value in treating immune-related diseases, such as A-T syndrome.

The innate immune response plays a key role in the etiopathogenesis of autoimmune diseases such as systemic lupus erythematosus (SLE), rheumatoid arthritis (RA), Sjogren’s syndrome (SS), systemic sclerosis (SSc) and Aicardi-Goutières syndrome (AGS). The etiological factors of SLE include genetic factors, environmental factors and hormonal factors [ 90, 91] . Notably, the production of type I IFNs is increased in SLE patients [ 92– 94] , and the severity of SLE has been reported to be dependent on the amount of type I IFNs [95], implying the fundamental role of inflammation in the onset of SLE. Endosomal TLRs play key roles in regulating type I IFN expression in autoimmune diseases, for example, TLR7 and TLR9 for SLE and TLR3 for SSc [ 96, 97] . Thus, the use of TLR antagonists might be a worthwhile strategy to consider for anti-IFN therapy for autoimmune diseases.

More interestingly, recent findings have also indicated that DNA repair deficiency and DNA damage accumulation can lead to apoptosis and autoantibody production in SLE patients [ 84, 98, 99] . In SLE, the γH2AX level is increased, and the expression of DNA repair factors such as PARP1, RAD50 and ATM is significantly reduced, indicating that DSB repair plays a crucial role in the pathology of SLE [ 84, 98, 100] . The cGAS–STING–type I IFN pathway is also activated in the skin of SLE patients [ 101, 102] . Thus, targeting both IFN signaling and the DSB repair pathway seems to be a promising approach to ameliorate the phenotypes of SLE.

AGS is a rare neurological disease with onset in infancy. AGS shares multiple symptoms similar to those of SLE, and the level of type I IFNs is also increased in AGS children. Three-prime repair exonuclease 1 (TREX1) plays a very important role in preventing the immune response by degrading cytosolic DNA fragments. *TREX1* mutations have been observed in both SLE and AGS patients, and cGAS is required for triggering the autoimmune response by inducing type I IFNs in these contexts [ 103– 105] . Understanding the dynamic regulation of the production, degradation and sensing of DNA fragments in AGS will improve our understanding of the origination and development of AGS and promote the development of novel approaches to treat AGS in the future.

Collectively, these findings suggest that antagonists of nucleic acid sensing pathways and activators of DNA damage repair pathways may be employed as novel therapeutic strategies for the treatment of immune-related diseases.

## Concluding Remarks and Perspectives

DNA damage repair and innate immunity are both evolutionarily conserved and ancient mechanisms involved in stress responses. Recently, a large body of research has demonstrated that a close relationship exists between these two biological processes. As we reviewed here, DNA damage can initiate immune signaling, and several classical factors participating in DSB repair have also been shown to regulate innate immunity. Conversely, nucleic acid sensors, including both DNA and RNA sensors, can also enter the nucleus to regulate both pathways of DSB repair. This evidence strongly implies that the two processes have much more complicated interactions than was thought in the past.

Aging in humans is characterized by a decrease in physical and mental capacity and an increased incidence of disease. It has been well recognized that DNA repair capacity declines and DNA damage accumulates with aging, and unresolved DNA damage initiates DNA damage response signaling, further activates the p53-mediated cascade, leads to apoptosis and senescence, and eventually contributes to the onset of aging and aging-related diseases. For more details on the relationship between DNA repair and aging, please refer to a recent review article [106]. In recent years, inflammation has been revealed to be another possible intermediate event among DNA damage, cell fate determination and aging-related degeneration. Moreover, inflammation can be further spread to induce DNA damage, impair DNA repair and influence the fate of nearby cells (which has been referred to as paracrine senescence). Consistent with this, chronic inflammation, which goes beyond the physiological threshold, has been shown to play a fundamental role in regulating aging and aging-related diseases and is therefore termed inflammaging [ 107, 108] . Targeting the senescence-associated secretory phenotype (SASP) with senomorphics, a class of small molecules that suppress the production and secretion of inflammatory factors without inducing apoptosis, also exhibits great promise in alleviating aging and chronic diseases [109]. Although these studies expand our understanding of the crosstalk between DNA repair, innate immunity and aging, the underlying mechanism and the roles of DNA repair and the immune response in the onset of aging and aging-associated diseases still require further investigation.

Intriguingly, several unique animal models provide valuable hints to help us understand the connections among innate immunity, DNA repair and longevity. Bats are mammals with great resistance to virus infection and show remarkable longevity (considering that bats have a similar size to mice). Bats carry multiple lethal viruses and live in large colonies, which facilitates virus transmission. Nevertheless, some species of bats have evolved robust immune responses to viruses. For instance, the S185 site of IRF3 [110], a potential phosphorylation site, and some DSB repair factors, including DNA-PK and RAD50 [111], which have also been reported to function as DNA sensors, are positively selected in some species of bats to enhance the antiviral function. On the other hand, bats have also evolved unique mechanisms, including downregulation of TNF-α expression and dampened activation of the NLRP3 inflammasome, to counteract excessive inflammation, which is one of the pillars of the biology of aging. For a thorough review of the regulatory mechanism of aging and virus toleration in bats, please refer to a recent review article [112]. Whether these features together contribute to the longevity of bats is an interesting topic to be explored. Retrotransposons are another source of genetic damage and have been found to promote inflammation in senescent cells and aged individuals [ 113, 114] . Recently, a study demonstrated that activation of retrotransposable elements results from cell hyperplasia, leading to the accumulation of cytoplasmic RNA:DNA hybrids and activating the cGAS–STING pathway, ultimately inducing cell death to prevent cancer formation in blind mole rats which are long-lived rodents [115]. Nevertheless, consistent with the antagonistic pleiotropy hypothesis, this mechanism may also cause sterile inflammation and accelerate the aging process in later life. Whether the accumulation of damaged dsDNA in the cytosol functions in a similar manner in regulating cancer formation and aging and whether this pathway exists in other species are also important directions for future studies.

Deficiency in either DNA repair or innate immunity leads to serious human diseases. Several studies have elucidated that genome instability triggers inflammation, possibly dependent on the activity of nucleic acid sensors, eventually giving rise to deleterious consequences, including embryo lethality, cardiovascular diseases, neurological dysfunction and progeria [ 116– 119] . Identifying the core molecules that induce the onset of these pathologies will lay the foundation for the development of novel treatments for these diseases in the future.
